# Role of ketogenic diet and its effect on the periodontium. A scoping review

**DOI:** 10.3389/froh.2024.1364578

**Published:** 2024-02-01

**Authors:** Hala Al Taher, Aya Salah, Caroline Rammal, Sudhir Rama Varma

**Affiliations:** ^1^ Department of Clinical Sciences, Ajman University, Ajman, United Arab Emirates; ^2^ Center for Medical and Bio-Allied Health Sciences Research, Ajman University, Ajman, United Arab Emirates

**Keywords:** ketogenic diet, ketone diet, oral health, periodontal health, periodontitis

## Abstract

The purpose of this study is to investigate the relationship between the ketogenic diet and periodontitis, as well as the nature of such relationship. Furthermore, emphasis was given to know whether ketogenic diet causes changes in oral health parameters and more specifically on periodontal health. Studies from 2010 to 2023 were reviewed and analyzed. Databases used to search included PubMed, Mednet, Scopus, Cochrane, and Embase. The literature reviewed was limited to randomized clinical trials, observational studies, and case-control studies. Of the eight studies included, three studies found that diets with similarities to the ketone-based diet could have a significant positive impact on periodontal health. One study pointed to the potential positive effect of a diet such as keto, but no definitive conclusion could be made. The current body of evidence concluded that there may be a relationship between keto and periodontitis, although the evidence is not consistent. It can be implied, however, that it is a positive relationship as ketogenic diet has been shown to have an anti-inflammatory effect, reducing inflammatory markers found in many diseases, including periodontitis.

## Introduction

With the aging population increasing due to advances in healthcare and living conditions, chronic inflammatory diseases such as periodontitis have also been on the rise. The prevalence of moderate to severe periodontitis among adults aged 65 and over was 47.2% (1). This is significant because periodontitis is a leading cause of tooth loss, which can cause both functional and psychological issues, affecting quality of life. Over the recent years, people have been making more effort to retain their teeth due to aesthetic and social reasons. Contrary to popular belief, periodontitis does not only affect the older population. Although prevalence varies due to bias, it affects 20%–50% of the global population, including developing and developed countries (2). In light of this, there is a need for increased awareness and education about the advent of periodontitis and the risk factors that contribute to it.

Periodontitis is a chronic and debilitating oral disease characterized by the inflammation and eventual destruction of the periodontal tissue, including the gingiva, alveolar bone, and cementum, which support the teeth (2). The disease is mainly caused by accumulated dental plaque and subsequent mineralization into calculus (tartar), which harbors pathogenic microorganisms. Periodontitis begins as gingivitis or gum inflammation, then forms pockets that progress apically to the alveolar bone. Around 800 species of bacteria are involved, producing many virulent toxins and enzymes that incite a chronic inflammatory response in the host, destroying the periodontal tissue and, if left untreated, tooth loss (2).

The etiology of periodontitis is multi-factorial and encompasses a range of risk factors, such as genetics, hormonal fluctuations, stress, and, most importantly, diet (3). Some of these factors are modifiable, including smoking, oral hygiene, and, to some extent, diet. These factors are worth looking into, as understanding their correlation with periodontitis can lead us to a step closer to prevention. Although a good deal of research has been conducted over the past few years regarding the relationship between smoking and periodontitis, little attention has been given to diet as a risk factor.

### Role of diet in the progression of periodontitis

**Figure 1:**
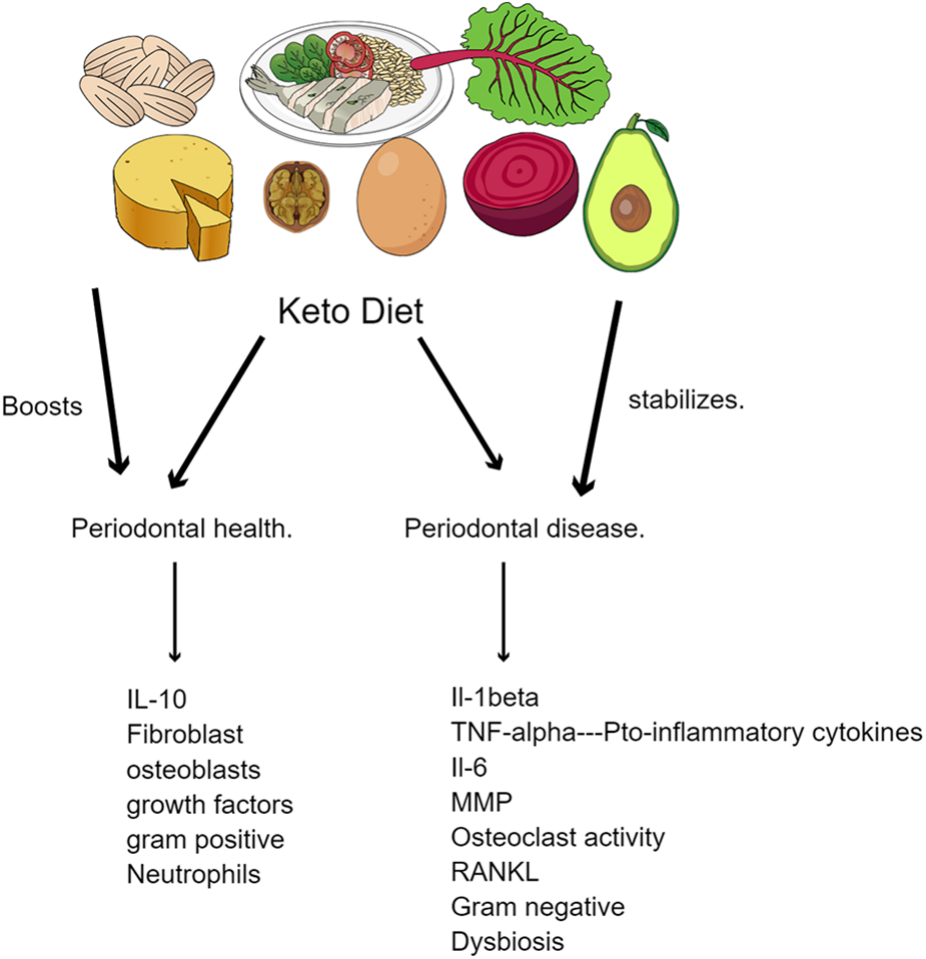
Role of ketogenic diet on periodontal disease progression.

Interestingly, diet plays a crucial role in the advent of periodontal disease since food can act as a substrate for the oral microbiota and provide them with a favorable environment to grow and multiply (3)*.* The oral microbiome is one of the most complex in the body, coming only second to the intestinal microbiota, and disruption of its harmony can promote periodontal disease (3). Our diet comprises micronutrients and macronutrients, which affect periodontal health (3, 4). Micronutrients, vitamins A, C, D, and E, and melatonin have antioxidant effects, slowing the inflammatory onset of periodontitis (4). As for macronutrients, these are required in large amounts and include minerals, carbohydrates, and fats. Studies have revealed a strong correlation between the incidence of periodontitis and dietary habits, particularly those high in refined carbohydrates and sugars. Oral microorganisms efficiently metabolize these dietary components into acid, lowering the oral cavity's pH. This leads to tooth enamel erosion and disruption of the oral microbiome, causing plaque formation and calculus accumulation. Processed foods, which have highly refined carbohydrates and are low in essential nutrients, have also been linked to the development of periodontitis (4, 5). These foods provide a substrate for oral microorganisms and contribute to nutrient deficiencies, hindering the ability of the host to produce a robust immune response to oral pathogens. It facilitates a bi-directional pathway whereby with the absence of essential nutrients, periodontal inflammation is seen with an increase in pro-inflammatory biomarkers such as IL-1β, IL-6 and Matrix metallo-proteinases and a biofilm which is dysbiotic. When essential nutrients are within acceptable limits it brings about the presence of predominantly synthetic cells contributing to periodontal health (Figure 1).

Conversely, a diet rich in omega-3, plant nitrates, and phytochemicals has been shown to protect against periodontitis (4). They are rich in vitamins and minerals, which support the host's immune system and provide a less favorable environment for oral pathogens (3, 4). This happens because dietary antioxidants can mitigate the harmful effects of inflammation through their ability to scavenge and neutralize free radicals, thereby reducing the risk of chronic inflammatory conditions such as periodontitis and maintaining cellular integrity. Free radicals cause oxidative damage to the gums and surrounding tissues. This effect can be reversed by consuming dietary antioxidants or reducing exposure to free-radical-generating sources such as tobacco and certain medications. These medications include chemotherapeutic, non-steroidal anti-inflammatory, anesthetic, and cardiovascular drugs (6). Phytochemical compounds found in plants have health-promoting properties, antioxidant, anti-inflammatory, and anticancer effects and have deterrent influence on pro-inflammatory activities (7). Vitamins A, C, and E have been linked to a reduced risk of periodontitis due to their antioxidant properties (4). In addition, a study following Danish students who incorporated whey protein, calcium, and vitamin D in their diet showed a reduced risk of severe periodontitis (7).

As for Carbohydrates, they are the body's primary source of energy and are crucial for proper function. Commonly used carbohydrate supplements include glucose, fructose, and maltodextrin. A diet that is high in sugar and processed carbohydrates can increase the risk of periodontal diseases. However, complex carbohydrates such as fruits and vegetables can help to maintain overall health and mitigate the risk of periodontal diseases (4). As public health awareness increases, people become more interested in leading a healthy lifestyle. With obesity reaching an all-time high, hypertension and type 2 diabetes are on the rise, which creates an urgency for both the management and the prevention of this epidemic. Various diets have emerged over the past few years, such as vegetarian, caloric restriction, and intermittent fasting. Diets such as vegetarian and Mediterranean diets emphasize the consumption of fruits, vegetables, and olive oil. As previously stated, such food groups have a lower risk of periodontitis (4).

However, today, it is considered one of the leading non-pharmacological therapies for the management of obesity (8, 9). A study on women following a very low-calorie ketogenic diet showed a significant reduction in weight, BMI, and waist circumference (10). The ketogenic diet consists of low carbohydrates (less than 10%), moderate protein, and high fat (8, 11). When the carbohydrates are reduced to less than 50 grams daily, the glycogen stores are depleted, and the body produces ketone bodies as an alternative energy source (8). Current evidence proves the ketogenic diet's efficacy in initiating and maintaining weight loss and its safety regarding the liver, thyroid, and kidney function in the long term (10).

Interestingly, the ketogenic diet has also positively affected cognitive function and cardiovascular risk factors (12). Additionally, the keto diet has been very popular with diabetic patients. Studies have linked keto with better glycemic control, probably due to the diet's reduced carbohydrate content (13). Diabetic patients who follow a ketogenic diet have been observed to experience weight loss, better-fasting glucose and insulin levels, lower cholesterol, and, in some cases, the ability to reduce or eliminate their diabetic medication (13). The ketogenic diet is still used as a therapeutic dietary approach to epilepsy today, as well as many other diseases, including polycystic ovary syndrome and even cancer.

On the other hand, the keto diet can have some potential harms and disadvantages. Potential harm is constipation due to a lack of fiber and fruits, vegetables, and whole grains (8). Diarrhea may also occur in some individuals due to the fat content present in the diet. Over time, a high protein and fat intake may also increase the risk of kidney stones (8). Other long-term issues associated with following the ketogenic diet include hepatic steatosis and hyperproteinemia. It is also worth mentioning that long-term adherence to the ketogenic diet is difficult, as it is pretty restrictive (8).

### Relationship between keto and periodontitis

Despite the extensive research, little to no data exists regarding the keto diet's effect on clinical oral parameters. The current data needs to be more consistent. A study concerning patients following an anti-inflammatory, low-carbohydrate, vitamin-rich diet showed a clinically significant decrease in gum inflammation and reduced bleeding on probing (5). Another study, a randomized clinical trial in which subjects were put on a diet low in carbohydrates and rich in fibers and vitamins C and D, showed similar results (7). Oral parameters such as plaque index, bleeding on probing, and pocket depth were cut in half (7).

Only some studies have reported that following a keto diet might result in disparity among commonly seen clinical oral parameters such as salivation, healing, and keratinization (12, 14). Keto diet may contain saturated fat, which leads to an increase in LDL cholesterol in normal-weight healthy women (12). Much evidence suggests a connection between periodontal diseases and high cholesterol levels in the body (14). This relationship between periodontitis and lipid levels is likely a result of the systemic effects of inflammation. According to another study, elevated levels of LDL cholesterol in the serum were linked to clinical loss of attachment and gingival plaque (14).

It remains unclear whether diets such as keto affect oral parameters. Therefore, we aim to determine whether the keto diet is beneficial or detrimental to oral health. In addition, we would like to investigate the relationship between the keto diet and the progression of periodontitis. We would also like to determine the strength of the correlation, if it exists, between the keto diet as a modifiable risk factor and periodontitis.

## Materials and methods

**Figure 2:**
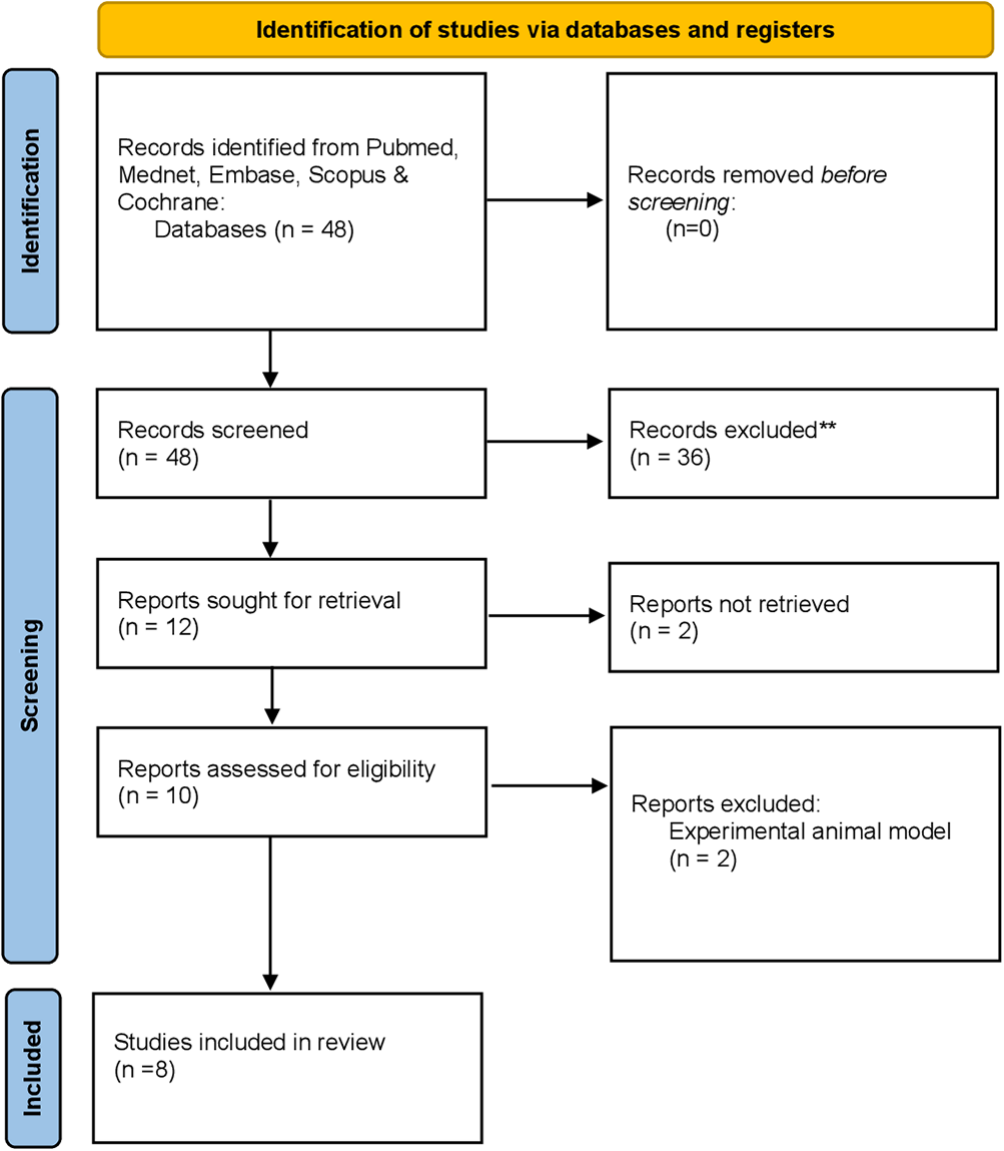
Flowchart highlighting the selection process of articles retrieved from digital databases.

Studies from 2010 to 2023 were reviewed and analyzed. Databases used to search included PubMed, Mednet, Scopus, Cochrane, and Embase. The keywords used for the search included ((ketogenic diet) OR (keto diet)) AND (oral health). When typing keto diet OR Ketogenic diet AND periodontitis, the keywords showed only one result. We wanted to evaluate articles that mentioned the keto diet's significance in periodontitis conditions in their clinical trials. Hence, we used a broader terminology. The literature reviewed was limited to randomized clinical trials, observational studies, and case-control studies. Articles merely listing the advantages or disadvantages of the diet were excluded. Articles in English language only were selected. Narrative and systematic reviews were excluded. The review contained ten articles that were thoroughly read and analyzed (Figure 2).

## Results

The present study investigates the relationship between the ketogenic diet and periodontitis. The relationship between diet and periodontal health has recently become a point of interest, with many interventional studies confirming this link. Of the eight studies included, only two directly investigated the potential relationship between keto and periodontitis (15–17). Another pilot trial found no changes in periodontal health in subjects after following a 6-week ketogenic diet (18). Three studies found that diets similar to the ketone-based diet could significantly positively impact periodontal health (16, 19, 20). These include two randomized clinical trials in which subjects following an oral-health-optimized diet containing reduced carbohydrates showed decreased potential periodontal bacterial species in the supragingival oral plaque and decreased periodontal inflammation (19, 20). One study pointed to the potential positive effect of a diet such as keto, but no definitive conclusion could be made (18). Another RCT is associated with using sugar substitutes such as erythritol, commonly used in the keto diet as sugar is restricted, with reduced plaque growth (21). One study showed that the ketogenic diet has an anti-inflammatory effect, which could be necessary as periodontitis is a chronic inflammatory disease (16, 20) (Table 1).

**Table 1:** List of clinical studies enumerating the role of ketogenic diet in periodontal disease progression.

Author	Year	Study type	Sample size	Results
Hesham El-Sharkawy, et al.	2010	Parallel design clinical study	80	Dietary supplementation with polyunsaturated fatty acids and aspirin results in probing depths reduction and a significant attachment gain after 3 and 6 months
Hoo-Seob Park, et al.	2015	Pilot study	53	Dietary change &weight control could reduce the amounts of periodontal biomarkers in GCF without periodontal intervention
J. P. Woelber, et al.	2016	Randomized controlled study	15	A diet low in carbohydrates, rich in Omega-3 fatty acids, rich in vitamins C and D, and rich in fibers can significantly reduce gingival and periodontal inflammation
Étienne Myette-Côté, et al.	2018	Randomized controlled study	11	Low calorie diet proved to be effective in reducing inflammation in type 2 diabetes
David M Shaw, et al.	2020	Randomized controlled study	8	Adaptation to a ketogenic diet led to a significant increase in T regulatory cells, and significant decrease in salivary IgA. Therefore, regulatory immune functions were stabilized
Johan Peter Woelber, et al.	2021	Exploratory pilot trial	20	The ketogenic diet did not lead to neither worsening or improvement in periodontal health but it did lead to weight loss
Christian Tennert, et al.	2021	Randomized controlled pilot study.	14	An oral health-optimized diet reduced the amount of potentially harmful oral bacteria in plaque, which may lead to improved oral health.
Riina Runnel, et al.	2016	Randomized controlled double-blind, three-arm parallel clinical trial.	Not-specified	Ketogenic diet could improve periodontal and gum disease condition along with low caries incidence

## Discussion

Like most chronic diseases, periodontitis can be influenced by many external factors. Research suggests that environmental factors, genetics, epigenetics, and lifestyle choices could contribute to the development of periodontitis by influencing the composition of the biofilm and the host's inflammatory immune response (22).

Periodontitis has been described as an inflammatory disease mainly resulting from the invasion of both the innate and the adaptive immune systems. The development of bacterial biofilm, sometimes called dental plaque, on the teeth's surface initiates the disease process. Pathogenic and commensal microorganisms coexist in the complex and dynamic community known as the bacterial biofilm. The expanding bacterial biofilm produces many virulence factors, including lipopolysaccharides, proteases, and toxins, and can start and maintain the inflammatory response in the periodontal tissues (22).

Neutrophils, macrophages, and T lymphocytes are just a few immune cells drawn to the infection site as part of the host response to the bacterial attack. The cytokines and chemokines these immune cells release increase the inflammatory response, activating osteoclasts and bone resorption (9). The periodontal fibroblast is one of the primary cells in the pathophysiology of periodontitis. These cells are the primary cell type in the periodontal ligament and are critical in maintaining periodontal tissue homeostasis. However, in periodontitis, pro-inflammatory cytokines, such as interleukin-1beta and tumor necrosis factor-alpha, can promote the destruction of the periodontal tissues (23).

In addition, osteoclasts play a significant role in periodontitis. The inflammatory cytokines released by immune cells and periodontal fibroblasts in periodontitis cause osteoclast activation. Osteoclast activation ultimately leads to the loss of alveolar bone, a defining feature of periodontitis (23). Furthermore, microbial colonization in periodontitis is a crucial part of the disease process in addition to the host response. Porphyromonas gingivalis, Treponema denticola, and Tannerella forsythia are a few examples of pathogenic bacteria linked to periodontitis and are thought to contribute to the development and spread of the condition (24). These microorganisms create virulence factors that can undermine host defenses and aid in the survival of the pathogenic biofilm.

Therefore, microbial colonization and host response interact in a complicated way during the pathogenesis of periodontitis. Once bacterial biofilm builds up, immune cells become activated, pro-inflammatory cytokines are produced, and osteoclasts become active, which causes periodontal tissues to be destroyed. To create successful therapeutic approaches to treat periodontitis, it is essential to understand the pathophysiology of the disease and identify the major immune cell types and bacteria involved in the disease process (24).

Although the relationship between the ketogenic diet and periodontitis remains mainly unexplored, understanding the mechanisms of this diet and its impact on other chronic diseases could bring us closer to identifying a correlation. A ketogenic diet exerts various effects on health, including metabolism, brain function, and cancer prevention through ketosis. The pathogenesis of the ketogenic diet involves the production of ketone bodies, which are produced in the absence of carbohydrates. These ketone bodies are metabolized in the liver when the body is in a state of low carbohydrate availability or increased fatty acid breakdown. The body produces small amounts of ketone bodies that can yield 22 ATP each in health (25). This highly efficient process helps the body meet its energy demands when glucose availability is limited. Ketone body production is regulated mainly by insulin, which inhibits ketogenesis when glucose levels are high (26). For this reason, the ketogenic diet has been indicated for diabetic patients—according to a randomized clinical trial, a low-carbohydrate, high-fat diet led to better glycemic control and reduced inflammation (20).

One potential therapeutic effect of the ketogenic diet is a reduction in inflammation. Chronic inflammation is a significant contributor to periodontal disease, and studies have shown that the ketogenic diet does reduce inflammation in the body. This suggests that the diet may have a protective effect on the periodontium. The ketogenic diet has been observed to reduce the expression of various pro-inflammatory markers. These include cyclooxygenase 2, nuclear factor-k, and macrophage inflammatory protein 2. C-reactive protein has been linked to various diseases. Few studies suggest a ketogenic diet may reduce CRP levels, indicating a potential anti-inflammatory effect. A study where a hypocaloric carbohydrate diet was consumed for 12 weeks found that the C-reactive protein levels had significantly lowered (20, 26). A study demonstrated that reducing dietary carbohydrate intake improved pro-inflammatory markers such as TNF-α, IL-6, and IL-8 (27). Another cross-sectional study evaluated the effect of a low carbohydrate diet on two inflammatory markers, IL-1β and Galectin-3. This study further proved that LCD reduced the level of these two inflammatory markers (28).

On the other hand, the ketogenic diet may have adverse effects on periodontal health. It may lead to a decreased production of saliva. Saliva is crucial due to its cleansing effect and neutralizing acids in the mouth. Low salivary production can lead to dry mouth, erosive activity, gum disease, and other oral health problems (19). Additionally, it was found that a high-fat diet can induce periodontitis in mice by increasing the expression of LPS receptors, leading to an inflammatory response (29). It is important to note that this was just a high-fat diet, which shares similarities with the ketogenic diet but differs in various aspects.

Our review identified several studies showing the ketogenic diet's anti-inflammatory effects. This is consistent with a review article published in 2022 by Srivastava et al. This article found that the ketogenic diet has immune-modulatory and anti-inflammatory effects and is therefore beneficial in various chronic inflammatory diseases ranging from polycystic ovary syndrome to cardiovascular diseases and diabetes, and in our case, periodontitis (30). Similarly, our review contained studies that reported reductions in inflammatory markers in individuals following a ketogenic diet, which may contribute to improved periodontal health. Moreover, another review conducted by Pinto et al. (2018) found that the ketogenic diet has antioxidant and anti-inflammatory properties (6). While these studies suggest a possible anti-inflammatory effect of the keto diet on periodontitis, it is essential to note that the current evidence is minimal, and more research is required to fully evaluate the relationship between the ketogenic diet and periodontitis. In addition, many other factors, such as lifestyle and genetics, influence an individual's response to dietary interventions and conditions such as periodontitis. Therefore, while promising results, caution must be taken during interpretation.

Overall, we explored the potential impacts of the ketogenic diet on the periodontium. While several findings suggested a positive anti-inflammatory effect, as discussed above, some articles suggested a negative impact, and some suggested no relationship. Alternatively, Martinon et al. emphasize the importance of nutrient-dense diets containing calcium, polyphenols, and vitamins, which may not necessarily be present in a ketogenic diet (31). Furthermore, they discuss the potential benefits of dietary interventions such as probiotics, prebiotics, and plant-based diets in improving periodontal health (31, 32). Although both reviews emphasize different aspects of diet, both suggest that diet plays a role in the progression of periodontitis. Additionally, both studies recognize the importance of nutrition as a modifiable factor in the advent of periodontal disease.

There are very few interventional studies that directly investigate the nature of the relationship between the ketogenic diet and periodontitis. The current evidence is promising if such a relationship exists due to the ketogenic diet's anti-inflammatory and anti-oxidative effects. However, some studies have reported a potential negative impact of the ketogenic diet due to its high fat content. It cannot be denied that nutrition does play a vital role as a modifiable factor in periodontitis, but the results of specific dietary interventions such as keto remain unknown. More interventional studies with a larger population are needed to come up with a definitive conclusion.

Few limitations exist and must be considered while examining the studies' findings. With the majority of studies describing the effect of keto on other health conditions, there needs to be more scientific research regarding keto and periodontitis. It is also important to note that it is challenging to isolate dietary interventions, making it difficult to establish a correlation. Moreover, more interventional studies need to be conducted. Additionally, there may be individual variations in response to the ketogenic diet, impacting its efficacy in enhancing periodontal health.

## Conclusion

Periodontitis has many factors that affect its progression, including lifestyle and nutrition. Growing evidence suggests that nutrition plays a crucial role in periodontitis. However, such a relationship is complex, and the evidence must be sufficient to use dietary interventions as the sole therapeutic approach to periodontitis. The current body of evidence concluded that there may be a relationship between keto and periodontitis, although the evidence is not consistent. It can be implied, however, that it is a positive relationship as the ketogenic diet has been shown to have an anti-inflammatory effect, reducing inflammatory markers found in many diseases, including periodontitis.

With the prevalence of periodontitis and the tremendous therapeutic potential of the ketogenic diet, this relationship cannot be ignored. More evidence is needed to confirm this correlation. Notably, more interventional studies with a larger population are needed to explore the ketogenic diet's effect on the periodontium properly.

## Data Availability

The original contributions presented in the study are included in the article/Supplementary Material, further inquiries can be directed to the corresponding author.
